# Introducing ORTO-R: a revision of ORTO-15: Based on the re-assessment of original data

**DOI:** 10.1007/s40519-020-00924-5

**Published:** 2020-05-20

**Authors:** Radosław Rogoza, Lorenzo M. Donini

**Affiliations:** 1 Institute of Psychology, Community-Based Mental Health Center for Children and Adolescent: Warsaw-Bielany District, Cardinal Stefan Wyszyński University, Wóycickiego 1/3, 01-938 Warsaw, Poland; 2 Sapienza University in Rome, Rome, Italy

**Keywords:** Orthorexia nervosa, Measurement, ORTO-R, ORTO-15

## Abstract

**Background:**

Orthorexia nervosa has attracted significant attention in the field, however, alongside increasing knowledge, more and more gaps are being identified. One of the fundamental problems concerns measurement of orthorexia nervosa. The most commonly used self-report measure, the ORTO-15, demonstrated an unstable factorial structure across different populations. Therefore, one might question whether the knowledge obtained from past research using ORTO-15 is valid or not. The aim of the present paper is to re-analyse original data used for the validation of ORTO-15 to assess its factorial structure and propose its revision, the ORTO-R.

**Methods:**

The description of the sample and procedure corresponds to the one reported in Donini et al. (Eat Weight Disord 10:28–32, 2005). *N *= 525 subjects were enrolled. To evaluate whether the factorial structure of ORTO-15, we used confirmatory factor analysis. The results revealed that the ORTO-15 indeed does not capture the structure of orthorexia nervosa adequately and revision is needed. The ORTO-R contains six items from ORTO-15, which were identified as the best markers of orthorexia nervosa.

**Discussion and conclusion:**

In the current paper, we present a refined measure of orthorexia nervosa—the ORTO-R. It is based on a frequently used ORTO-15, overcoming its main limitations. We strongly believe that the current work will act as a bridge, linking past with the future research, and that alongside a new measure, the field of research on orthorexia nervosa will move forward.

**Level of evidence:**

Level V, descriptive study.

## Introduction

Orthorexia nervosa (ON) can be described as an obsession with eating healthy and proper food [1]. As reviewed by Cena et al. [2], what the diagnostic criteria proposed by multiple researchers [3–6] all have in common are the following characteristics: (1) obsessive preoccupation with healthy nutrition, (2) behaviours that includes rigidly following a restrictive ‘healthy’ diet with strict avoidance of foods believed to be unhealthy and impure, (3) violations of their diet result in extreme emotional distress with feelings of guilt and anxiety, (4) psychosocial impairments in social and vocational/academic functioning, and (5) physical impairments related to nutritional deficiencies. Although the research on ON is currently flourishing, there are also many significant limitations. For example, there are no official diagnostic criteria of ON present in the Diagnostic and Statistical Manual for mental disorders (DSM; [7]), and its status as a disorder is unclear as well (i.e., whether it should be considered as distinct disorder, a variant of eating or obsessive compulsive disorder or as a disturbed eating habit [8]). Thus, to move the field of ON research forward, in 2016, the Orthorexia Nervosa Task Force was established. One of its main goals (see [2]) is to validate a new self-administered questionnaire, starting from ORTO-15. Within the current paper, we realise this goal through re-assessment of the original data reported in the ORTO-15 validation paper [9].

Within the field of ON research, the majority of studies rely on ORTO-15 developed by Donini and colleagues [9] (see [2] for comprehensive review); however, its measurement quality is frequently questioned as, for instance, it yields different results across different population groups [10]. Its development was clearly a milestone in our understanding of ON as it certainly moved the field forward, however, there are also a few limitations of the measure, which make researchers doubt in the accuracy of their results. The problems could be grouped into two major problems and some other more specific limitations: assessment of ON prevalence and the unstable factorial structure of the ORTO-15.

ORTO-15 was proposed as a test for the diagnosis of ON, whose prevalence was assessed to be approximately 6.9% [9]. It should be noted that the diagnosis presented in the text [9] was also based on the simultaneous presence of a selective (not necessarily healthy) diet and high level of obsession (based on the MMPI). Although that would be a reasonable number for a self-report measure, whose main goal is to screen rather than to diagnose, subsequent studies reported that ORTO-15 diagnoses (without taking into consideration the presence of selective diet and high levels of obsession) ON in up to 88.7% of participants (nutrition students [11]). Only a few studies replicated the original findings, suggesting the ON prevalence to be around 6% [12, 13], however, the majority of other findings suggest the prevalence exceeds 50% [14, 15]. Although some ORTO-15 items might be seen not as a measure of pathology but rather of healthy eating, one should note that they mostly focus on attitudes not necessarily correct in the choice of food to eat. Therefore, it is not surprising that ORTO-15 was criticised for being inaccurate [4]. This is especially harmful for research on ON, as when it is being diagnosed, taking into account behavioural symptoms and internal states, its prevalence is approximated to be less than 1% [16]. All these findings suggest that ORTO-15 as a self-report measure is unable to distinguish between healthy and pathologically healthy eating and, therefore should not be used as a diagnostic measure alone.

ORTO-15 was not constructed in the spirit of classical test theory, and almost no psychometric data accompanying the original publication [9]. This study aims to fill this gap, using original data to provide desperately needed psychometrical evidence for ORTO-15. To date, there are six national adaptations of ORTO-15 into German, Hungarian, Polish, Portuguese, Spanish and Turkish [10, 17–21]. In these studies, there are versions which advocate existence of one [21], two [19] or three factors [18]. The study on the German population [10] provided evidence that neither the one-factorial nor three-factorial [9] models are found in the data. Moreover, although each of the national versions used the same version of ORTO-15 [9] each single study retained different items, that is—there are no two equivalent versions of the ORTO-15 questionnaire. Recent meta-analysis of these studies [22], which is in high congruence to empirical studies examining the structure of ORTO-15 [20, 23–27], revealed, however, that six items, which all load onto one factor of ON, are common to all these national adaptations, creating a promising starting point for further research.^1^

One of the most recent critical papers on the measurement of ORTO-15 [27] reports that ORTO-15’s factor structure has an unacceptable model fit and low internal reliability and that research on ON should use a different measure. While we agree that this statement is accurate when regarding the full ORTO-15, in our opinion, it is rather a call for refinement of the measure rather than its complete removal from the literature. Actually, the findings from Meule et al. [27], support the meta-analytical suggestion [22] that six items might be better indicators as the strength of the factor loadings was consistently highest of all analysed items. Modification of scoring might not only change the internal structure of the ORTO measure, however, it might also elucidate findings, which would be otherwise missed. For example, [28] using mixed methodology of interviews and self-report, used ORTO-15 and applied traditional scoring as well as summating six items identified in meta-analysis [22]. Within the study [28], assessed factors which might contribute to progress towards unhealthy preoccupation with healthy eating. They found that orthorexic thoughts and behaviours might be triggered by the desire to have a fit and thin body, but this was relevant only when they considered abbreviated version of the measure.

These six items partially taps the main characteristics [2]. More specifically, item ‘Does the thought about food worry you more than three hours a day?’ assesses obsessive preoccupation with healthy nutrition (criterion 1). Item ‘Are your eating choices conditioned by your worry about your health status?’ implies that the person does try to eat healthily (criterion 2). Item ‘Do you think that consuming healthy food may improve your appearance?’ potentially might also assess criterion 2, however, in its current form, it is rather a mild indicator. Items ‘In the last three months, did the thought of food worry you?’ and ‘Does the thought about food worry you for more than three hours a day?’, and ‘Do you think that the conviction to eat only healthy food increases self-esteem?’ regards emotional distress and feelings of guilt, and anxiety (criterion 3). Finally, the item ‘Do you think that eating healthy food changes your lifestyle (frequency of eating out, friends, …)?’ regards psychosocial impairments in social functioning (criterion 4). The final criterion (i.e., physical impairments related to nutritional deficiencies) is not covered by these items.

The sources of these problems lie within the early development of the ORTO-15 [9] as the initial pool of items was not the subject of any factor analytic procedure and were only validated. This resulted in a situation where weak items that should be discarded during the questionnaire development are still present. Although the factorial validity of the ORTO-15 is questionable, its content validity needs further evaluation as it might be an indicator of healthy eating instead of pathological dieting. Although the internal consistency of ORTO-15 was not reported in original study [9], the estimates of internal consistency range from 0.14 to 0.83 (*M* = 0.55; SD = 0.27 [29]), suggesting that the items comprising the measure are related only to limited extent.

Moreover, there is a number of specific limitations. First of all, ORTO-15 was developed prior to recognised definitions for ON [3–6]. Second, ORTO-15 items were based on Bratman’s book presenting an American ideal, which might not suit the Italian population. However, given the unstable factorial structure of the ORTO-15, it is nearly impossible to conduct tests of measurement invariance, which would make such cultural comparisons more meaningful. Third, the scoring of the ORTO-15 is counter intuitive, where low or even medium scores indicate higher pathology. Summing up, ORTO-15 have many weaknesses that need to be addressed. And although we are unable to address all of them at once, we believe that ORTO-15 needs refinement. Therefore, in the current study, we propose a solution, the ORTO-R, which: (1) is created in the sense of the classical test theory; (2) has a stable factorial structure; (3) is a promising measure for conducting measurement invariance analysis; (3) DOES NOT assess the prevalence of ON; and (4) makes the administration of the measure more appealing and intuitive.

### Current study

In the light with the problems associated with the measurement of ON by ORTO-15, we question whether past data provided accurate results. Using original data used for the development of ORTO-15 [9], we aim to verify the factorial structure and to evaluate the extent to which past data yielded accurate results. Given the difficulties with establishing a stable factorial structure in national adaptations, we expect that neither (H1A) the one-factorial nor (H1B) three-factorial model will be well fitted to the data. In contrast, given the findings from meta-analytic investigation of the ORTO-15 structure [22], we expect that it is possible to retain such items, which would capture the structure of ON as one factor, which we propose to label ORTO-R. Finally, we expect (H3) that the results obtained from past data using original ORTO-15 are important and valid, therefore, we expect that the revised version will be positively correlated at the threshold of collinearity (i.e., > f0.80 [30]). Finally, to provide further evidence of the ORTO-R validity, we analysed the proposed measurement model using data from Missbach et al. [10], who criticised the measurement model of ORTO-15. We expected that our proposition will solve problems with factorial validity in the German population [10].

## Method

### Participants and procedure

The description of the sample and procedure corresponds to the one reported in Donini et al. [9]. The participants were selected at the Sapienza University between February and August 2001. Enrolment of volunteers and the collection of data were both carried out by trained persons with appropriate knowledge of Food Science and in Research on Eating Behaviour. In sum, *N *= 525 subjects were enrolled in this way. Spontaneous enrolment gave us subjects with various different occupational characteristics: employees came from the Institute of Biochemistry of the Sapienza University, from the Ministry of the Italian Air Force, from the television channel Sat 2000; students enrolled from the Plinio Scientific High School and from the Sapienza University; parents of children in the 4th class of the San Giuseppe Junior School and parents of patients attending the Pediatric Dietetics Service at the Umberto I Hospital in Rome; a group of residents from Frosinone, near Rome, etc. Subjects under the age of 16 were excluded, because they were considered insufficiently autonomous in the choice of their food. Further information about sample could be found in Donini et al. [9]. The missing data did not exceed 0.8% on any item.

### Statistical analyses

To evaluate whether the factorial structure of ORTO-15, we used Confirmatory Factor Analysis (CFA). To evaluate model fit we relied on standard recommendations, that is, the values of Comparative Fit Index (CFI) should be greater than 0.900 and the values of Root Mean Square Error of Approximation (RMSEA) should be lower than 0.080 [31]. As ORTO-15 comprise only four response options, we treated the data as categorical [32] and all of the analyses were carried out on polychoric correlation matrices using Weighted Least Squares with Mean and Variance Adjusted (WLSMV) estimation method in Mplus v.7.2 [33]. Given the used estimation, we also report the estimates of the weighted root mean square residual (WRMR), which values lower than 1 suggest good fit to the data [34]. In the assessment of reliability of measurement, because of the limitations associated to coefficient *α* (e.g., assumption of equal factor loadings and residuals; [35], we report coefficient *ω* [36], which provides more accurate results [37]. All the used scripts are available at the https://osf.io/qfuz5.

## Results

First, we tested whether the one- and three-factorial (comprising cognitive-rational, clinical, and emotional factors; [9] model represent good fit to the data. As hypothesised, the results of CFA, which standardized factor loadings are presented in Table 1 suggested that the data was poorly fitted to the tested one—(*χ*_(90)_^2^ = 474.01; *p* < 0.001; CFI = 0.689; RMSEA = 0.090[0.082, 0.098]; WRMR = 1.73) and three-factorial (*χ*_(87)_^2^ = 438.61; *p* < 0.001; CFI = 0.715; RMSEA = 0.088[0.080, 0.096]; WRMR = 1.66) model.

**Table 1:** Standardized Factor Loadings of the One- and Three-Factorial Measurement Model of ORTO-15

Item content	One-factorial	Three-factorial
1. When eating, do you pay attention to the calories of the food? (R)	**0.27**	**0.27**
2. When you go in a food shop do you feel confused? (R)	− 0.45	− 0.43
3. In the last 3 months, did the thoughts of food worry you?	0.57	0.70
4. Are your eating choices conditioned by your worry about your health status?	0.48	0.46
5. Is the taste of food more important than the quality when you evaluate food? (R)	**− 0.01**	**− 0.04**
6. Are you willing to spend more money to have healthier food?	**0.30**	**0.33**
7. Does the thought about food worry you for more than three hours a day?	0.47	0.50
8. Do you allow yourself any eating transgressions? (R)	**0.05**	**0.09**
9. Do you think your mood affects your eating behaviour? (R)	0.46	0.55
10. Do you think that the conviction to eat only healthy food increases self-esteem?	0.62	0.59
11. Do you think that eating healthy food changes your lifestyle (frequency of eating out, friends, …)?	0.57	0.62
12. Do you think that consuming healthy food may improve your appearance?	0.60	0.68
13. Do you feel guilty when transgressing? (R)	**0.39**	**0.37**
14. Do you think that on the market there is also unhealthy food?	**0.31**	**0.33**
15. At present, are you alone when having meals?	**0.17**	**0.21**

(R) = reversely coded item. In three-factorial model, following items were assigned to factors: cognitive-rational (1, 5, 6, 11, 12, 14), clinical (3, 7, 8, 9, 15), and emotional (2, 4, 10, 13). Values below 0.40 are presented in bold

In both tested models, out of 15 items, the strength of the standardised factor loadings of four items (1, 5, 8, and 15) was lower than 0.30, and of three (6, 13, 14) was lower than 0.40. Moreover, one of the items (2) had opposite direction. Therefore, over half of the original ORTO-15 items appeared to be poor indicators in terms of factorial validity. All the remaining seven items seem to be valid indicators of ON. However, given the fact that only one item (9) is reversely coded and caused problems in national adaptations [10, 19], for the sake of brevity, we argue for the exclusion of this item from the revised version. Therefore, we propose to label the remaining six items (3, 4, 7, 10, 11, and 12), also identified in the meta-analysis [22] as ORTO-R.

**Fig. 1:**
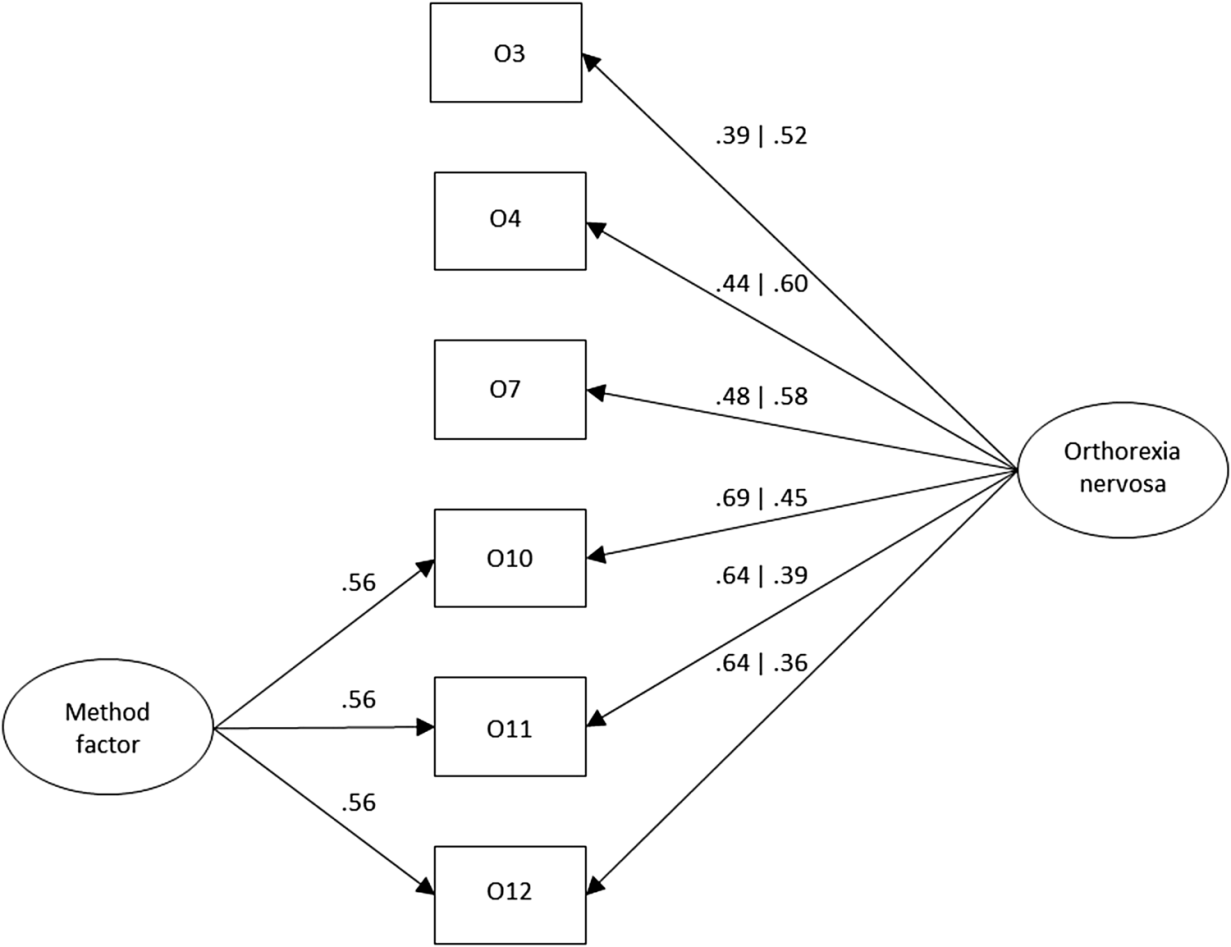
Standardized Factor Loadings of the Measurement Model of ORTO-R. Values before | corresponds to the model without method factor

We evaluated whether the ORTO-R as conceptualised as a one-factorial measure of ON fits to the data well. The results of CFA yielded ambiguous results (*χ*_(9)_^2^ = 65.06; *p* < 0.001; CFI = 0.900; RMSEA = 0.109[0.085, 0.135] WRMR = 1.21), which were mostly at the boundaries of acceptable thresholds. Therefore, we examined for potential sources for this lack of good model fit. Three of the ORTO-R items (10, 11, and 12—see Table 1) all begin with ‘Do you think …’ which might cause that participants to use a different responding style, providing artificial results [38]. To test the magnitude of this bias, we added to the measurement model an orthogonal latent factor loaded by three items which factor loadings were constrained to be equal (therefore, assuming that the effects of method bias were essentially the same on all selected test positions). The introduction of the method factor, significantly improved model fit, which was now good (*χ*_(8)_^2^ = 21.49; *p* = 0.006; CFI = 0.976; RMSEA = 0.057[0.028, 0.086], WRMR = 0.68), confirming the hypothesis on the underlying factorial structure.^2^ The standardised factor loadings of both tested ORTO-R models are provided in Fig. 1.

The method factor accounted for 31% of the variance (i.e., squared factor loading of method factor) captured by the items, therefore, its effects can be considered strong. The reliability of measurement of the ORTO-R was acceptable (*ω* = 0.75). Finally, we correlated the total score of the ORTO-R to the total score of ORTO-15 to see the extent to which these two versions are comparable. The one-tailed Pearson’s correlation revealed that they exceed the threshold of collinearity (*r* = 0.83; *p* < 0.001) and, therefore, confirming the last hypothesis.

The results from the data provided by Missbach et al. [10] were in high congruence to those reported above. That is, the analysed measurement model was slightly below acceptable thresholds (*χ*_(9)_^2^ = 194.62; *p* < 0.001; CFI = 0.894; RMSEA = 0.142[0.125, 0.159]; WRMR = 2.03), but alongside the introduction of the method factor, the model fit improved to a significant extent (*χ*_(8)_^2^ = 30.29; *p* < 0.001; CFI = 0.987; RMSEA = 0.052[0.033, 0.072]; WRMR = 0.74). The method factor accounted for 32% of variance, again suggesting its strong influence on the measurement quality. The strength of factor loadings was similar to those reported above, which were, respectively: 0.51, 0.53, 0.52, 0.64, 0.70, and 0.68 for the measurement model without the method factor and 0.65, 0.68, 0.59, 0.39, 0.43, and 0.43 for the measurement model in which we controlled for the influence of the method factor. Thus, our results appear consistent, confirming our expectations and providing further support for ORTO-R.

## Discussion

The goal of the current study was to evaluate the factorial structure of the most popular measure of orthorexic thoughts and behaviours—the ORTO-15 using original dataset used for the development of this instrument [9]. Lack of this statistical procedure resulted in a series of problems in most of the national adaptations investigating the factorial structure. Researchers were forced to remove items which appeared to be poor indicators of orthorexic thoughts and behaviours, and the measurement models differ from country to country, making any cross-cultural comparisons of orthorexic thoughts and behaviours impossible. Therefore, our goal was to revise the ORTO-15 and propose a stable factorial solution that could be applied in future research. We believe that this would catalyse future research on ON and limit the misuses within the field. Below, we discuss how our findings relate to past and future research.

### How the current findings contribute to the past research

In this study, we found that both the one- and three-factorial proposition [9] is poorly fitted to the data. Some of the test items (e.g., 1, 5, 8, and 15) did not measure orthorexic thoughts and behaviours (i.e., they remained uncorrelated to other items). Given this fact, difficulties found in national adaptations [10, 17–21] are understandable. Therefore, we see removal of some of the items as a necessary element for revising the ORTO-15 questionnaire. Our abbreviated proposal comprises six items that were found across all national adaptations [22]. The proposed measurement model was well fitted to the data, albeit not without some problems that needs to be addressed in future studies. However, as our approach seems to be a promising alternative in assessing orthorexic thoughts and behaviours, there is a need to re-evaluate existing national adaptations of ORTO-15 to see whether the ORTO-R also works in other countries as we see that most of these adaptations were heavily biased. We re-analysed data from the German adaptation [10] and demonstrated that although the authors heavily criticised the factorial structure of ORTO-15, when our proposition was taken into account, the measurement appeared to be much more precise. Therefore, the introduction of ORTO-R is likely to solve criticism around the measurement of orthorexic thoughts and behaviours [27].

The ORTO-15 was used in many studies, providing empirical illustration [6] that, for example, that orthorexic thoughts and behaviours^3^ are positively associated with eating disorders [39], that there are no gender differences in the levels of orthorexic eating behaviours [16, 40], that orthorexic eating behaviours are highly prevalent among eating disorders patients [13], that the orthorexic thoughts and behaviours are more common in the overweight and obese young people [41], and that they are associated with higher Instagram use [42]. In the light of our findings, these results might be questioned, therefore, we evaluated the extent to which our proposed model is related to ORTO-15. Both versions were correlated above the threshold of collinearity (i.e., >0.80), thus past findings might be seen as valid. There is no need to re-evaluate all of the past data using our refined model and existing systematic reviews [2] can be deemed accurate. Of course, such high correlation might also mean that ORTO-R ‘inherited’ weaknesses of its former version. At this point, however, we are unable to answer this question, and further work is needed.

### How the current findings contribute to the future research

As stated above, the proposed measurement model, although well fitted, had some internal problems that need to be addressed. The introduction of the method factor revealed that one-third of the variance was explained by systematic bias of the method (i.e., three out of six items began with ‘Do you think …’). Therefore, we argue that the wording of these items should be adjusted to eliminate this source of measurement error. Our proposal for how to solve this limitation is presented in the new ORTO-R, which is available to download on the OSF project page. Alongside the questionnaire, we provide an automated R-code which conducts confirmatory factor analysis and assesses reliability using ω coefficient.

The ORTO-15 was frequently used for the assessment of prevalence [4], which frequently provided biased results, that is, many studies suggested that the prevalence of ON is above 50% [11, 14, 21], whilst the real prevalence is approximated at no more than 1% [16]. Therefore, even in the light of the fact that some items were identified as weak indicators of orthorexic thoughts and behaviours, we believe that these results were harmful for the research on ON and we argue that ORTO-R should not be used for the assessment of prevalence. Instead, we advocate the dimensional approach introduced in DSM-5 [7]. Adopting such an approach in the research on ON increases the chance of including it within the future mental disorders classifications.

In light of the latter, we also argue that there is a need to re-specify the responding scale of ORTO-15 in two ways. First, we argue that the response scale should be reversed so the higher score on the questionnaire means higher levels of orthorexic behaviours. Such coding is typically used in the assessment of normal and pathological personality traits [43, 44] as well as many of other clinically relevant constructs such as eating disorders [45], depression [46] and self-esteem [47]. Although this is not a substantial change, reversal coding of the response scale in ORTO-15 resulted in a series of mistakes or awkward interpretations within the papers [24]. We believe that this cosmetic change will increase the comprehensiveness of the obtained results. Second, we argue that there is a need to introduce a five-point response scale. The current four-point scale requires treating the data as categorical [32], which results in more complicated statistical procedures (e.g., it requires replacement of the Pearson’s correlations by polychoric correlations). Because none of the national adaptions used appropriate statistical procedures, and therefore, provided biased results, we believe that this change will make the interpretation of the results easier.

The introduction of the ORTO-R creates a new opportunity for cross-national assessment of orthorexic thoughts and behaviours. Although there have been some attempts [48] they were methodologically limited as the national versions differed both in terms of items and underlying factorial structure [9, 19]. Thus, as a consequence, it was impossible to assess the measurement invariance, which is a standard statistical procedure used in cross-national comparisons [49]. We believe that the ORTO-R will overcome this limitation, and therefore, we call for cross-national studies comparing the orthorexic thoughts and behaviours across the world.

## Limitations

The ORTO-R was based on ORTO-15 [9]. Therefore, it shares some of its limitations (e.g., ORTO-15 was developed prior to the establishing typical characteristics of ON [2], it, therefore, does not fully correspond to all criteria). More specifically, its items do not correspond to the physical impairments related to nutritional deficiencies. Within the literature, there is, however, only one recently published and promising scale, which appropriately address this criterion [50]. To overcome some of these limitations, we reduced the original item pool from fifteen to six, and modified the retained item content to reduce the effects of identified method bias and simultaneously to increase their relevance to the characteristics of ON. The introduced changes are presented below, but also we provide a refined questionnaire template, and necessary R syntaxes (i.e., computing CFA and omega coefficient) at the OSF project site. Although we see ORTO-R as a promising measure, it still has to be validated in future studies, as we did not analyse the validity of the introduced changes to a significant extent. In the current paper, the ORTO-R already raised several questions. Although strong correlation between ORTO-R and ORTO-15 might be seen as a bridge connecting past research with new findings, such a correlation might also mean that our modifications did not increase the validity of the ORTO-R in comparison with its parent measure. When we controlled for the effects of method bias, the strength of the factor loadings decreased. While this could change in response to the introduced modifications, this has to be a subject of another study. Furthermore, we have found that the two-factor model represented almost an exact fit to the model with the method factor, which might mean that ORTO-R would actually be composed of two factors. In the current paper we opted for a unidimensional model as it is more parsimonious, however, future studies should assess whether our reasoning is reflected in new data. Finally, the estimates of internal consistency were not very convincing. This could be partially influenced by the fact that ORTO-R comprises only a few items, which might result in an underestimation of obtained internal consistencies in future studies, which is why we recommend reporting the omega coefficient. Again, this could potentially be solved through the introduced amendments to the item content, however, this issue needs to be addressed as well. In summary, ORTO-R at the current stage of its development faces some challenges, however, we believe that future studies would be able to solve them in favour of the refined measure of orthorexic thoughts and behaviours.

### What is already known on this subject?

ORTO-15 is one of the most popular measures of orthorexic thoughts and behaviours. However, numerous studies have noted its possible weaknesses such as an unstable factorial structure. These studies suggest removing some of the items; however, to date, there are at least six different proposals of which items should be removed and no commonly approved version of this measure exists.

### What does this study add?

Within the current paper we presented a refined measure of orthorexic thoughts and behaviours—the ORTO-R. It is based on a frequently used ORTO-15 [9], overcoming its main limitations. We strongly believe that the current work will act as a bridge, linking past with future research, and that alongside a new measure, the field of research on ON will move forward.

**Item Content of the ORTO-R**
1. Are your rigid and restrictive dietary choices conditioned by your worry about your health status?
2. Would you agree that eating healthy food increases your self-esteem?
3. Do you believe that strict consuming only of healthy food may improve your appearance?
4. In the last three months, did thoughts of food make you feel guilt, ashamed and anxious?
5. Does thinking about food excessively worry you for more than three hours a day?
6. Does eating healthy food change your lifestyle (frequency of eating out, friends, …)?

## Footnotes

1 Although in the Spanish adaptation one of the selected items was not present (item 10), its removal was not based on any statistical criteria.

2 We also tested an alternative hypothesis stating that the poor model fit was not due to the method bias, but because of the presence of the two factors. However, the results were equivalent in terms of fit indices (χ_(8)_^2^ = 22.96; *p* = 0.003; CFI = 0.973; RMSEA = 0.060[0.032, 0.089], WRMR = 0.70), therefore the more parsimonious one-factorial model seems to better represent the structure of ORTO-R.

3 Scores from ORTO-15 are negatively related to eating disorders pathology because of the reversed response scale in different measures, however, the association between ON and eating disorders pathology as constructs is positive.
